# Applying logistic LASSO regression for the diagnosis of atypical Crohn's disease

**DOI:** 10.1038/s41598-022-15609-5

**Published:** 2022-07-05

**Authors:** Ying Li, Fanggen Lu, Yani Yin

**Affiliations:** 1 Department of Infectious Diseases, Hunan Key Laboratory of Viral Hepatitis, Xiangya Hospital, Central South University, Changsha, 410013 China; 2 Department of Gastroenterology, The Second Xiangya Hospital of Central South University, Changsha, 410008 China; 3 Department of Gastroenterology of Xiangya Hospital, Central South University, Changsha, 410013 China; 4 Hunan International Scientific and Technological Cooperation Base of Artificial Intelligence Computer Aided Diagnosis and Treatment for Digestive Disease, Changsha, 410013 China; 5 National Clinical Research Center for Geriatric Disorders, Xiangya Hospital, Changsha, 410008 China

**Keywords:** Gastroenterology, Risk factors, Signs and symptoms

## Abstract

In countries with a high incidence of tuberculosis, the typical clinical features of Crohn's disease (CD) may be covered up after tuberculosis infection, and the identification of atypical Crohn's disease and intestinal tuberculosis (ITB) is still a dilemma for clinicians. Least absolute shrinkage and selection operator (LASSO) regression has been applied to select variables in disease diagnosis. However, its value in discriminating ITB and atypical Crohn's disease remains unknown. A total of 400 patients were enrolled from January 2014 to January 2019 in second Xiangya hospital Central South University.Among them, 57 indicators including clinical manifestations, laboratory results, endoscopic findings, computed tomography enterography features were collected for further analysis. R software version 3.6.1 (glmnet package) was used to perform the LASSO logistic regression analysis. SPSS 20.0 was used to perform Pearson chi-square test and binary logistic regression analysis. In the variable selection step, LASSO regression and Pearson chi-square test were applied to select the most valuable variables as candidates for further logistic regression analysis. Secondly, variables identified from step 1 were applied to construct binary logistic regression analysis. Receiver operating characteristic (ROC) curve analysis was performed on these models to assess the ability and the optimal cutoff value for diagnosis. The area under the ROC curve (AUC), sensitivity, specificity, positive predictive value (PPV), negative predictive value (NPV), accuracy rate, together with their 95% confidence and intervals (CIs) were calculated. MedCalc software (Version 16.8) was applied to analyze the ROC curves of models. 332 patients were eventually enrolled to build a binary logistic regression model to discriminate CD (including comprehensive CD and tuberculosis infected CD) and ITB. However, we did not get a satisfactory diagnostic value via applying the binary logistic regression model of comprehensive CD and ITB to predict tuberculosis infected CD and ITB (accuracy rate:79.2%VS 65.1%). Therefore, we further established a binary logistic regression model to discriminate atypical CD from ITB, based on Pearsonchi-square test (model1) and LASSO regression (model 2). Model 1 showed 89.9% specificity, 65.9% sensitivity, 88.5% PPV, 68.9% NPV, 76.9% diagnostic accuracy, and an AUC value of 0.811, and model 2 showed 80.6% specificity, 84.4% sensitivity, 82.3% PPV, 82.9% NPV, 82.6% diagnostic accuracy, and an AUC value of 0.887. The comparison of AUCs between model1 and model2 was statistically different (P < 0.05). Tuberculosis infection increases the difficulty of discriminating CD from ITB. LASSO regression showed a more efficient ability than Pearson chi-square test based logistic regression on differential diagnosing atypical CD and ITB.

## Introduction

Crohn's disease (CD) is a transmural inflammatory disease, which can affect the entire digestive tract from the mouth to the anus. In the early years, Crohn's disease was prevalent in western countries. However, with ethnic migration and the improvement of people's living standards, the incidence of Crohn's disease in developing countries, especially China, with an incidence increased from 0.28/100,000 in 1950–2002 to 0.848/100,000 in 1950–2007^1^. China has the second highest incidence of tuberculosis (TB) in the world. As a TB highly endemic country and considering the increasing incidence of CD, TB infection may cover up the typical appearance of CD. So it is called atypical CD, which is more difficult to distinguish from tuberculosis and also the typical CD^2,3^.

**Figure 1:**
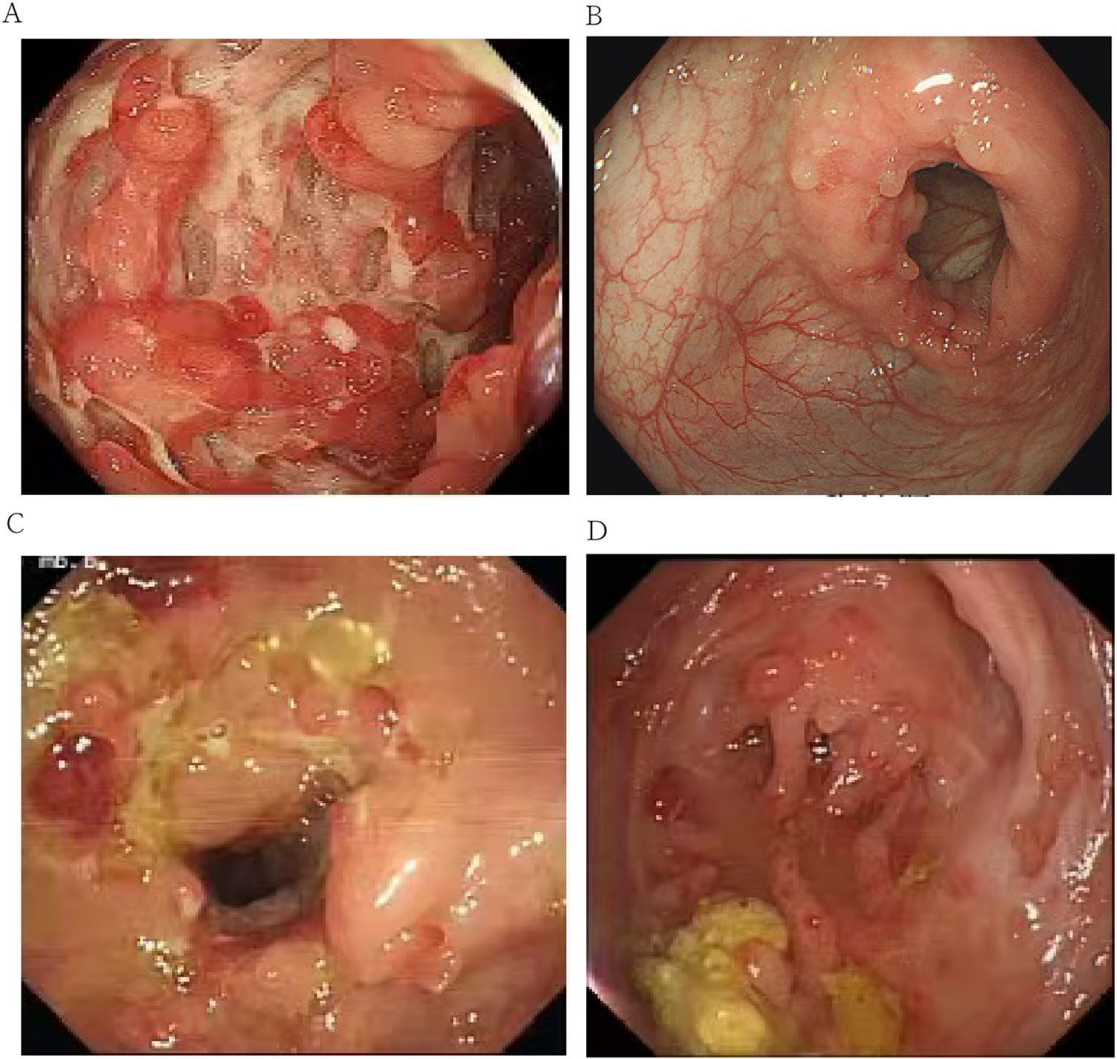
Different endoscopic appearances of CD and ITB.(**A**)typical cobblestone appearance in patients with CD. (**B**).transverse ulcer in patients with ITB. (**C**)CD patient associated with TB infection. (**D**) CD like patient with lymph node liquefaction,finally diagnosed ITB.

Though CD and ITB have different etiologies, there are many clinical, radiological, endoscopic manifestations overlapped (Fig. 1), especially in epidemic ITB areas, where the incidence of CD is increasing^4,5^. Acid fast bacilli (AFB) and granulomas with caseous necrosis are identified as golden criteria for diagnosing ITB, however, its value is limited with a positive rate at about 50%^6^. Generally, most of the studies report diarrhea, hematochezia, perianal disease, presence of longitudinal ulcers, aphthous ulcers, cobblestoning, and skip lesions are more common in CD, whereas presence of transverse ulcers and patulous ileocaecal valve are more common in ITB^7,8^. However, there is no gold standard for the diagnosis of CD. Clinicians make a decision largely relying on a comprehensive analysis of clinical, radiological, endoscopic manifestations. Nowadays, with the help of guide consensus, most Chinese patients with CD or ITB with typical disease characteristics obtain correctly and timely treatment. Gastroenterologists have also proposed multiple methods to improve the diagnostic value of the two diseases. He et al. applied random forest to screen variables and built two regression models based on 7 differential variables^9^. Wu et al. used t test and chi-square test to select variables and proposed a predictive model to discriminate CD and ITB^10^. Mao et al. established a model through univariate and multiple logistic regression analyses based on clinical and computed tomographic enterography (CTE) characteristics^11^. Models built via those methods were convenient and reliable for the differentiation of part of CD and ITB patients. Unfortunately, a study reported a misdiagnosis rate of the two disease was still at 50–70%^12^. Based on this, The methodology for the identification of these two diseases still needs to be further explored.

In clinical practice, anti-tuberculosis treatment was often used to discriminate the two disease. However, if patients with CD use anti-tuberculosis drugs, it will delay the course of the disease and bear the side effects of anti-tuberculosis drugs. Conversely, if ITB patients receive a treatment of CD, it will cause the spread of tuberculosis. In our previous study, a novel grouping strategy was already presented to separate CD into typical CD (TCD) and atypical CD (UCD). We proposed the phenotype of UCD is deeply influenced by TB infection history, which is also a major reason of misdiagnosis. we enrolled 141 atypical CD and ITB patients and built some predictive models based on clinical, radiological, endoscopic manifestations. However, we have not systematically and comprehensively explored the identification of CD and ITB, especially for the identification between atypical CD and ITB. Also, when identifying with ITB, we did not verify that different phenotypes of CD need to be discussed separately^13^. Therefore, more comprehensive information need to be defined when come across analysis of clinical phenotypes.

Conventional methods, such as stepwise selection, are usually applied for variable selection in diseases^14–18^. However, they have the disadvantage of overfitting^19^. LASSO (least absolute shrinkage and selection operator) is a variable selection method proposed by statistician Robert Tibshirani in 1996^20^.Compared to traditional regression approaches, LASSO regression can handle a larger set of potential predictors, picking out the variables most associated with the disease. Based on this, LASSO has been utilized in the screening of disease risk factors and establishment of prediction models^21–25^.Few studies have used LASSO regression to explore risk factors for CD^26–28^. However, to our knowledge, in the identification of CD and ITB, especially in the identification of atypical CD and ITB, whether LASSO regression can help differentiate the two diseases remains unknown.

In this study, we enrolled 332 CD and ITB patients, including 118 typical CD, 109 atypical CD and 105 ITB patients. We wish to deepen the understanding of the identification between CD and ITB and reduce the rate of misdiagnosis of the disease.

## Materials and methods

### Participants

This retrospective study was approved by the Ethics Committee of second Xiangya hospital Central South University, Changsha, China. All subjects provided written informed consent. All methods were performed in accordance with the relevant guidelines and regulations.

**Figure 2:**
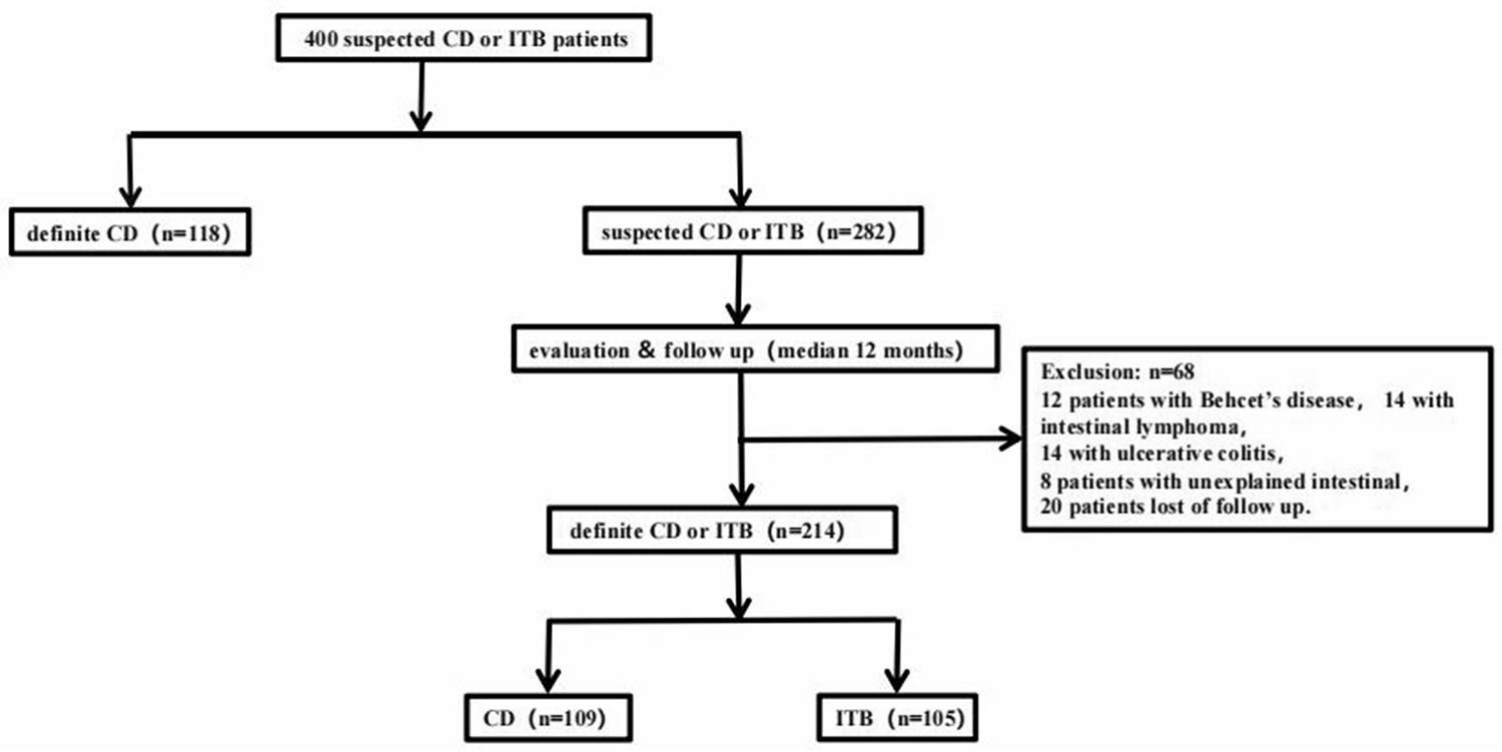
Flowchart for patients enrollment.

A total of 400 patients were enrolled from January 2014 to January 2019 in second Xiangya hospital Central South University. After a year-follow-up, 68 patients were excluded, including 14 patients with intestinal lymphomas, 12 patients with Behcet's disease, 14 patients with ulcerative colitis, 8 patients with unexplained intestinal ulcer, and 20 patients lost of follow up. Finally, we selected 332 patients to conduct our study, with 105 patients diagnosed with ITB and 227 patients diagnosed with CD. Among CD patients, 109 patients were defined as untypical CD (UCD), and 118 CD patients were defined as typical CD (TCD). UCD means the CD patient does not exclude tuberculosis; TCD means the CD patient excludes tuberculosis. Whether a patient with CD has tuberculosis infection is evaluated by a specialist. Detailed information was showed in Fig. 2.

### Inclusion criteria and exclusion criteria

The inclusion criteria were as follows: (1) Patients older than 18 years of age; (2) Patients were initially suspected of CD or ITB based on clinical data. The exclusion criteria were as follows: (1) ITB patients with caseous granuloma detected in pathological section when enrolled in the study; (2) Patients with HIV infection; (3) Patients diagnosed with other disease in the follow-up such as intestinal lymphomas, ulcerative colitis, Behcet's disease; (4) Patients lost of follow up.

### Data collection and definition

57 indexes were collected in this study, including demographic and clinical features, laboratory features, computed tomography enterography features, endoscopic features and site of involvement. All the continuous variables like albumin, platelets were changed into categorical variables. The detailed information was shown in supporting information (Table S1). Comprehensive assessment of T-SPOT, PPD and Lung CT results to assess the type of CD. If the assessment result does not exclude tuberculosis, then UCD is considered; if the result excludes tuberculosis, then TCD is considered.

### Diagnostic criteria and follow-up

Patients diagnosed with ITB should meet the following criteria: Patients who received regular anti-tuberculosis treatment at least 3 months and diagnosed with ITB after a-year follow-up according to clinical, laboratory, radiological, endoscopic features. Patients diagnosed with CD should meet at least 1 of the following criteria: (1) Noncaseating granuloma detected in pathological section during follow-up; (2) Patients who received regular immunosuppressive or biological agents at least 3 months and diagnosed with CD after a- year follow-up according to clinical, laboratory, radiological, endoscopic features; (3) Patients who received anti-tuberculosis drug treatment for 3 months, but did not get better after re-examination, and got better after receiving immunosuppressive agents or biological agents after a- year follow-up according to clinical, laboratory, radiological, endoscopic features.

### Statistical analysis

SPSS 20.0 and R software version 3.6.1 and the “glmnet” package (R Foundation for Statistical Computing, Vienna, Austria) were used for statistical analyses. The prediction model was developed using a 2-step approach. Firstly, Pearson chi-square test was used to compare enumeration data. Statistical significance was determined as a *P* value of less than 0.05. All variables with statistical significance (P < 0.05) in Pearson chi-square test was taken as candidates for further binary logistic regression analyses. We also used the LASSO regression to select the most valuable variables. The feature selection step was performed on complete data. Secondly, variables identified from step 1 were applied to constructed a binary logistic regression model. The regression coeffcients of the predictive model were regarded as the weights for the respective variables, and the score for each patient was calculated. Receiver operating characteristic (ROC) curve analysis was performed on these models to assess the ability and the optimal cutoff value for diagnosis. The area under the ROC curve (AUC), sensitivity, specificity, positive predictive value (PPV), negative predictive value (NPV), accuracy rate, together with their 95% confidence and intervals (CIs) were calculated. MedCalc software (Version 16.8) was applied to analyze the ROC curves of models.

## Results

### Univariate analysis for differentiation of CD and ITB

#### Univariate analysis for differentiation of comprehensive CD and ITB

We performed univariate analysis of 57 indicators including demographic characteristics, clinical characteristics, laboratory findings, imaging characteristics, and endoscopic characteristics between comprehensive CD and ITB, which was evaluated by Pearson chi-square analysis on 332 CD and ITB patients. 29 indicators were statistically different (P < 0.05, Table S2).

#### Univariate analysis for differentiation of UCD and ITB

Of the 57 indicators, 18 indicators were statistically different which were evaluated by Pearson chi-square test on 214 UCD and ITB patients. Among them, abdominal pain, diarrhea, perianal abscess, perianal fistula, ileus, bowl resection history, elevated platelets, decreased albumin, elevated CRP, FOBT positive, comb sign, segmental distribution of lesion, cobblestone appearance, longitudinal ulcers, jejunal involvement, rectal involvement were seen more frequently in CD, whereas positive PPD skin test and positive T-SPOT were more frequently identified in ITB (P < 0.05, Table 1).

**Table 1:** Clinical features of patients with UCD and ITB.

Variables	UCD (n = 109)	ITB (n = 105)	*P* value
Abdominal pain (%)	95.4	81.0	0.001
Diarrhea (%)	42.2	28.6	0.037
Perianal abscess (%)	11.9	2.90	0.012
Perianal fistula (%)	13.8	1.00	0.000
Ileus (%)	26.6	11.4	0.005
Bowl resection history (%)	15.6	6.67	0.038
Platelets↑ (%)	42.2	27.6	0.025
Albumin↓ (%)	83.5	70.5	0.024
CRP↑(%)	75.2	54.3	0.001
PPD skin test positive (%)	36.7	56.2	0.004
T-SPOT positive (%)	49.5	81.0	0.000
FOBT positive (%)	67.9	54.3	0.041
Comb sign (%)	13.8	0.00	0.000
Segmental distribution of lesion (%)	26.6	12.4	0.009
Cobblestone appearance (%)	14.7	0.90	0.000
Longitudinal ulcers (%)	24.8	8.60	0.002
Jejunal involvement	11.0	3.80	0.045
Rectal involvement (%)	31.2	19.0	0.041

**Figure 3:**
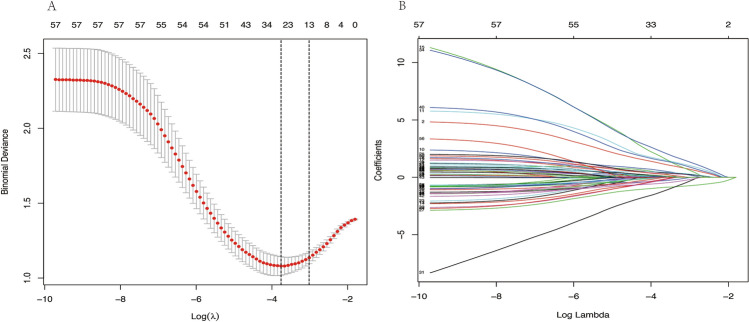
(**A**,**B)** LASSO regression showed log(λ) =  − 3.662 when the error of the model is minimized, and 26 variables were selected for further logistic regression analysis.

Since we have many variables and relatively few cases, to pick out the variables most associated with UCD and ITB, LASSO regression was applied to filter the variables on 214 UCD and ITB patients. We utilized ten-fold cross-validation to select the penalty term, lambda(λ). log(λ) =  − 3.662 (λ = 0.000217771) when the error of the model is minimized, and 26 variables were selected for further logistic regression analysis (Table S1, Fig. 3A,B).

### Development of a predictive model for differentiation between comprehensive CD and ITB

To establish a predictive model, we divided these 332 patients randomly into a training set (70%) and a validation set (30%). The training set comprised 236 cases (69 ITB patients and 167 CD patients), and the validation set comprised 96 cases (36 ITB patients and 60 CD patients). All variables with statistical significance (P < 0.05) selected by univariate analysis were taken as candidates for further logistic regression analyses. Based on binary logistic regression analysis, the regression was set as

$${\text{P}} = {1}/\left[ {{1} + {\text{e}}^{{ - (0.{884} - {1}.{\text{666X1}} + {3}.0{\text{43X2}} + 0.{\text{977X3}} + {1}.{\text{979X4}} + 0.{\text{861X5}} + {1}.{13}0{\text{X6}} - {2}.{\text{421X7}} - {1}.{\text{517X8}} + {3}.0{\text{13X9}})}} } \right]$$

(X1, night sweat; X2,perianal fistula; X3, illeus; X4, bowl resection history; X5, elevated PLT; X6, elevated CRP; X7, positive T-SPOT; X8, transverse ulcer; X9, involvement of jejunum). The cutoff value was 0.738, when P > 0.738, it was diagnosed with CD; when P < 0.738, it was diagnosed with ITB. The AUC, sensitivity, specificity, PPV, NPV, and accuracy rate of the prediction model were 0.907, 78.4%, 89.9%, 94.9%, 63.2% and 81.8%, respectively (Table 2). This predictive model was validated using the validation data set. ROC analysis showed that the AUC of the predictive model was 0.832 (95%CI, 0.750–0.915). With a cutoff value of 0.738, The sensitivity, specificity, PPV, NPV, and accuracy rate were 80%, 77.6%, 85.7%, 70% and 79.2%.

**Table 2:** Validation value of predicting model in differentiation between UCD and ITB.

Data set	Sensitivity (%)	Specificity (%)	NPV (%)	PPV (%)	Accuracy (%)
Development set	78.4	89.9	63.2	94.9	81.8
Validation set	77.8	55.6	56.8	76.9	65.1

Cutoff point for predictable diagnosed as ITB < 0.738.

Development set was randomly selected from 70% samples of comprehensive CD and ITB.

Validation set consists of 27 UCD and 36 ITB patients.

#### Validation data set from UCD and ITB patients

To validate the diagnostic value of the predictive regression model, we applied a validation data set of UCD and ITB patients (27UCD and 36 ITB patients), which was selected from the 96 cases of mentioned validation set. With a cutoff value of 0.738, the sensitivity, specificity, PPV, NPV, and accuracy rate were 77.8%, 55.6%, 56.8%, 76.9% and 65.1% in differentiating ITB from UCD (Table 2).

### Development of a predictive model for differentiation between UCD and ITB

Since we did not get a satisfactory diagnostic value via applying the regression model of comprehensive CD and ITB to predict UCD and ITB. We tried to explore a predictive model for the differentiation between UCD and ITB. In the first step, we applied all the 214 UCD and ITB patients to select features, using Pearson chi-square test (for model1) and LASSO regression (for medel2) respectively. In the second step, 214 UCD and ITB patients were randomly divided into a training set (70%) and a validation set (30%). Two binary logistic regressions were established with the variables selected by Pearson chi-square test (for model 1) and LASSO regression (for model 2), using the same training set (Table 3).We applied a validation data set of UCD and ITB patients to validate the efficacy of the two models (Table 4). Model 1 for diagnosing CD at the cutoff value of 0.665 showed 69.4% specificity, 66.67% sensitivity, 62% PPV, 73.5% NPV, and 68.3% diagnostic accuracy; and with the cutoff value of 0.421, model 2 showed 75.8% specificity, 56.2% sensitivity, 69.2% PPV, 64.1% NPV, and 66.2% diagnostic accuracy. By statistically analyzing the ROC curves of the two models using MedCalc software (Version 16.8), we got a P value of < 0.05. This result indicated that the diagnostic value of model 2 was better than model 1.

**Table 3:** Diagnostic equations of prediction models in differentiation between ITB and UCD.

Differential diagnosis	Equations
Model 1	P = 1/[1 + e^−(−1.806+1.882X1+3.037X2+1.290X3+0.973X4–1.319X5+2.374X6+2.047X7)^] X1, abdominal pain; X2, perianal fistula; X3, illeus; X4, elevated CRP; X5, T-SPOT positive; X6, cobblestone appearance; X7, involvement of jejunum
Mode2	P = 1/[1 + e^−(−1.488+1.524X1+2.408X2+1.250X3–1.633X4–1.305X5+1.306X6–1.692X7+1.332X8+1.513X9+2.071X10–2.027X11+1.457X12+2.647X13)^] X1, abdominal pain; X2, perianal abscess; X3, illeus; X4, hepatobiliary disease; X5, tuberculosis history; X6, eleveted CRP; X7, T-SPOT; X8, segmental lesions; X9, longitudinal ulcer; X10, jejunum involvement; X11, ascending colon involvement; X12, rectum involvement; X13, perianal fistula

Model 1: Pearson chi-square test based logistic regression; Model 2: LASSO regression based logistic regression.

**Table 4:** Comparison between model 1 and model 2.

Model	Sensitivity (%)	Specificity (%)	NPV (%)	PPV (%)	Accuracy (%)	AUC^a^	95%CI lower	95%CI upper	P value
Model1	65.9	89.9	68.9	88.5	76.9	0.811	0.743	0.879	0.000
Model2	84.4	80.6	82.9	82.3	82.6	0.887	0.835	0.939	0.000

Model 1: Pearson chi-square test based logistic regression; Model 2: LASSO regression based logistic regression.

^a^AUCs between model 1 and model 2 were statistically different (P < 0.05).

### Development of a predictive model for differentiation between UCD and TCD

Univariate analysis was also performed between TCD and UCD. Interestingly, 16 indicators were statistically different (P < 0.05, Table S3). This prompted us to further explore the predictive equations that could identify these two phenotypes. 227 CD patients were randomly divided into a training set (70%) and a validation set (30%). The training set comprised 164 cases (82 TCD patients and 82 UCD patients), and the validation set comprised 63 cases (36 TCD patients and 27 UCD patients). All variables with statistical significance (P < 0.05) selected by univariate analysis were taken as candidates for further binary logistic regression analysis. Based on binary logistic regression analysis, the regression was set as

$${\text{P}} = {1}/\left[ {{1} + {\text{e}}^{{ - ( - {2}.{62}0 + {1}.{\text{633X1}} + {1}.{\text{563X2}} + 0.{\text{899X3}} + 0.{\text{888X4}} + {1}.{3}0{\text{6X5}} - 0.{\text{995X6}} - {1}.{\text{354X7}} + 0.{\text{972X8}})}} } \right]$$

(X1, bowl resection history; X2, intestinal wall edema; X3, intestinal stenosis; X4, increased fat density; X5 shallow ulcer; X6 swollen ileocecal valve; X7 involvement of ileum; X8 involvement of sigmoid colon). The cutoff value was 0.66, when P > 0.54, it was diagnosed with TCD; when P < 0.54, it was diagnosed with UCD. The AUC, sensitivity, specificity, PPV, NPV, and accuracy of the prediction model were 0.825, 80.5%, 70.7%, 78.4%, 73.3% and 75.6%, respectively. This predictive model was validated using the validation data set. The sensitivity, specificity, PPV, NPV, and accuracy rate were 47.2%, 52.9%, 60.7%, 45.7%,and 52.3%.

## Discussion

Due to many overlapping symptoms, CD and ITB are difficult to distinguish. In recent years, with the increasing incidence of CD in China, physicians have gradually deepened their understanding of CD and formed a consensus on the diagnosis of CD. Under the guidance of consensus, most typical CD patients were correctly diagnosed. However, for those CD patients accompanied by latent tuberculosis infection, though the guidelines recommend diagnostic anti-tuberculosis treatment for identification, it may not an optimal choice. Based on this, our study was the first time to systematically demonstrate the differentiation between CD and ITB, we clarified that tuberculosis infection increased the difficulty of discriminating CD from ITB,and CD patients under different circumstances need to be considered separately when distinguishing from ITB.

There are multiple studies on the identification of CD and ITB^11,29–31^. Jung et al.^30^ built a seven-marker model to discriminate CD and ITB, with a sensitivity, specificity of 98.0, 92.4, respectively. Their study enrolled 261 patients and indicated that age, sex, ring shape ulcers, suspicious pulmonary tuberculosis, diarrhea, longitudinal ulcers, and involvement of the sigmoid colon were the important indexes for discrimination. We also created a predictive model for the same purpose. However, compared to Jung’s study, our model showed 9 indicators including night sweat, perianal fistula, intestinal obstruction, intestinal surgery, elevated PLT, elevated CRP, T-SPOT positive, transverse ulcer, involvement of jejunum were the vital markers to differentiate the two diseases,with a sensitivity, specificity of 78.4%, 89.9%, respectively. Although we have a larger sample size, our results have a lower diagnostic efficacy. We suspected that TB infection may affect the CD phentype as times change, making it more difficult to identify the two disease.

With the help of guidelines as well as the multivariate mathematical models, most typical CD patients could receive a correctly and timely treatment. However, there is still a high rate of misdiagnosis for CD when distinguishing from ITB. We speculated the reason is that previous studies did not consider typical CD and atypical CD separately. These methods were not applicable to the discrimination between ITB and atypical CD patients. To verify this, in this study, we substituted the UCD and ITB data into the previously established model, and found that the diagnostic deficiency was getting worse as we suspected, with accuracy rate decreased from 81.8 to 65.1%. This finding prompted us to look for ways to identify atypical CD and ITB. Until now, few studies were carried out to differentiate UCD from ITB. Zhao et al.^32^ pointed that the level of tuberculosis interferon gamma release assay (TB-IGRA) could help discriminate the UCD and ITB, and if TB-IGRA ≥ 100 pg/ml, the possibility of ITB should be considered first, and diagnostic anti-TB treatment should be recommended. However, previous TB infection history may lead to false positives in TB-IGRA results. Due to the small sample size of this study, the conclusions need to be verified. Our previous research also explored the distinction between UCD and ITB. We enrolled 43 UCD and 56 UITB patients, and built 4 regression equations from clinical, laboratory, endoscopic, and radiological features, respectively^13^. Though AUC of clinical prediction model was 0.834, the model is not suitable for the application in clinical practice due to the subjectivity of its variable collection. Hence, in this study, we comprehensively analyzed the clinical, laboratory, endoscopic, and radiological features of UCD and ITB. We used Chi-square test and LASSO regression to filter variables. Results of the two methods revealed abdominal pain, perianal fistula, ileus, elevated CRP, cobblestone appearance, involvement of jejunum were favor of UCD, while T-SPOT positive were favor of ITB, which indicated these three indexes were reliable for the identification of the the two disease. We constructed two equations to predict the two disease, with the AUC over 80%. though these two equations had a partial missed diagnosis rate, they still provided a valuable method for the identification of UCD and ITB. It is worth mentioning that LASSO regression was considered to be a better method to choose variables when the size of predicted variables were relatively large^33^, our results showed the diagnostic value of training set in model 2 was prior to model 1, however, it is not much different when comparing the validation set, which may be related to the sample size.

Our research further analyzed the characteristics of typical CD and atypical CD. Results showed that swollen ileocecal valve, involvement of ileum ulcer were favor of UCD, while intestinal surgery, intestinal wall edema, intestinal obstruction, blurred fat gap, shallow ulcer involvement of sigmoid colon were favor of TCD. Most of these indicators come from radiological examination, which suggested that when we diagnose CD, we should pay attention to the results of radiological examination, which may help reduce misdiagnosis. We also noticed that the indicators that support the diagnosis of UCD were similar to those of intestinal tuberculosis mentioned in previous studies^34–36^. We are not surprised because China is a country with a high incidence of tuberculosis, and the phenotype of UCD may be affected by tuberculosis infection to some extent. We further established the regression equation based on the indexes obtained by the multivariate regression analysis. Interestingly, we found that the two different subgroups of CD can be identified by the equation, with the AUC, sensitivity, specificity, PPV, NPV, and accuracy of the prediction model were 0.825, 80.5%, 70.7%, 78.4%, 73.3% and 75.6%, respectively. The diagnostic value was similar to that of the discrimination between UCD and ITB.This indicated that in the identification of ITB and CD, it is necessary to consider tuberculosis-infected CD separately,especially in areas where tuberculosis is epidemic.

In conclusion, there were several advantages in our study. Firstly, we proposed a new method to distinguish between CD and ITB, and we confirmed that identification of UCD and ITB required additional attention,especially in areas with a high incidence of tuberculosis,the existence of UCD affected the misdiagnosis rate of CD and ITB that should not be ignored. Secondly, we constructed two equations for the discrimination between UCD and ITB, and we were the first to apply LASSO regression to select variables and build a model for the identification of the two diseases. Thirdly, we constructed a equation to discriminate TCD and UCD, this validated that when discriminating between ITB and CD, we should consider UCD and TCD separately. Our research has enriched the identification methods of CD and ITB, and provided valuable guidance for clinical practice. However, there are also some limitations. This study is a single-center research and lack of an independent external dataset for testing the models. Although LASSO regression helps us to select and confirm some identification of parameters, these parameters may not be widely used due to geographical differences, multi-center data needs to be collected. In the future, to reduce the misdiagnosis rate of CD, new biomarker exploration is needed. Other methods such as neural networks and random forests can also be used in larger data sets to build predicted models. In epidemic ITB areas, efforts are still needed to increase the detection rate of Mycobacterium tuberculosis.

## Supplementary Information

Supplementary Information.

## Data Availability

The datasets generated and/or analysed during the current study are not available since we are still collecting more data for further study, but are available from the corresponding author on reasonable request.
