# Supplementary material for: Applying logistic LASSO regression for the diagnosis of atypical Crohn's disease
Source: Sci Rep. 2022 Jul 5;12:11340. doi: 10.1038/s41598-022-15609-5 (PMC9256608; doi:10.1038/s41598-022-15609-5)
Supplement: Supplementary file 1 — Supplementary Information. [file 41598_2022_15609_MOESM1_ESM.zip › Supplementary information/tableS2.doc]

**TableS2.Clinical features of patients with comprehensive CD and ITB**

| **variables** | **CD (n=227)** | **ITB (n=105)** | ***P* value** |
| --- | --- | --- | --- |
| Gender (%)  Male  Female | 79.3  20.7 | 68.6  31.4 | 0.034 |
| Diarrhea (%) | 46.7 | 28.6 | 0.002 |
| Night sweat (%) | 8.40 | 16.2 | 0.033 |
| Weight loss (%) | 55.1 | 41.0 | 0.017 |
| Perianal abscess (%) | 15.0 | 2.90 | 0.001 |
| Perianal fistula (%) | 18.5 | 1.00 | 0.000 |
| Ileus (%) | 30.4 | 11.4 | 0.000 |
| Bowel perforation (%) | 7.50 | 0.00 | 0.004 |
| Tuberculosis history (%) | 4.40 | 18.1 | 0.000 |
| Bowl resection history (%) | 24.7 | 6.70 | 0.000 |
| Platelets↑(%) | 45.8 | 27.6 | 0.000 |
| Albumin↓ (%) | 83.7 | 70.5 | 0.005 |
| CRP↑(%)  PPD skin test positive (%)  T-SPOT positive (%)  FOBT positive (%)  Bowel wall thickening (%)  Ascites (%)  Intestinal stenosis (%)  Comb sign (%)  Increased small mesenteric vessels (%)  Segmental distribution of lesion (%)  Cobblestone appearance (%)  Transverse ulcer (%)  Longitudinal ulcers (%)  Cecal involvement (%)  Jejunal involvement (%)  Sigmoid colon involvement (%)  Rectal involvement (%) | 75.8  17.6  23.8  68.3  92.1  0.40  48.0  13.7  38.3  27.3  12.3  8.80  30.0  15.0  12.3  42.7  38.3 | 54.3  56.2  81.0  54.3  78.1  5.70  36.2  0.00  20.0  12.4  0.90  20.0  8.60  23.8  3.80  28.6  19.0 | 0.000 0.000  0.000 0.014  0.000 0.007  0.044 0.000  0.001  0.002 0.001  0.004 0.000  0.050 0.014  0.014  0.000 |
