# Supplementary material for: Applying logistic LASSO regression for the diagnosis of atypical Crohn's disease
Source: Sci Rep. 2022 Jul 5;12:11340. doi: 10.1038/s41598-022-15609-5 (PMC9256608; doi:10.1038/s41598-022-15609-5)
Supplement: Supplementary file 1 — Supplementary Information. [file 41598_2022_15609_MOESM1_ESM.zip › Supplementary information/tableS3.doc]

**TableS3. Clinical features of patients with atypical CD and typical CD**

| **Variables** | **TCD (n=118)** | **UCD (n=109)** | ***P* value** |
| --- | --- | --- | --- |
| Abdominal pain (%)  Night sweat (%)  Tuberculosis history (%)  Bowl resection history (%) | 78.8  2.50  0.00  33.1 | 95.4  14.7  9.20  15.6 | 0.000  0.001  0.002  0.002 |
| PPD positive (%)  T-SPOT positive (%)  Bowel wall thickening (%) | 0.00  0.00  97.5 | 36.7  49.5  86.2 | 0.000  0.000  0.002 |
| Intestinal stenosis (%)  Increased fat density（%） | 57.6  33.9 | 37.6  16.5 | 0.003  0.003 |
| Increased small mesenteric vessels (%)  Transverse ulcer (%)  Shallow ulcer (%)  Swollen ileocecal valve (%)  Ileum involvement (%)  Sigmoid colon involvement (%)  Rectal involvement (%) | 48.3  4.20  23.7  19.5  12.7  50.8  44.9 | 27.5  13.8  11.0  32.1  27.5  33.9  31.2 | 0.001  0.011  0.012  0.029  0.005  0.010  0.034 |
